# The *Cryptococcus gattii* species complex: Unique pathogenic yeasts with understudied virulence mechanisms

**DOI:** 10.1371/journal.pntd.0010916

**Published:** 2022-12-15

**Authors:** Lamin Saidykhan, Chinaemerem U. Onyishi, Robin C. May

**Affiliations:** 1 Institute of Microbiology & Infection and School of Biosciences, University of Birmingham, Edgbaston, Birmingham, United Kingdom; 2 Division of Physical and Natural Science, University of The Gambia, Brikama Campus, West Coast Region, The Gambia; Albert Einstein College of Medicine, UNITED STATES

## Abstract

Members of *Cryptococcus gattii/neoformans* species complex are the etiological agents of the potentially fatal human fungal infection cryptococcosis. *C*. *gattii* and its sister species cause disease in both immunocompetent and immunocompromised hosts, while the closely related species *C*. *neoformans and C*. *deneoformans* predominantly infect immunocompromised hosts. To date, most studies have focused on similarities in pathogenesis between these two groups, but over recent years, important differences have become apparent. In this review paper, we highlight some of the major phenotypic differences between the *C*. *gattii* and *neoformans* species complexes and justify the need to study the virulence and pathogenicity of the *C*. *gattii* species complex as a distinct cryptococcal group.

## Introduction

*Cryptococcus gattii* (*sensu stricto*) was first recognized and described as a distinct cryptococcal strain from *Cryptococcus neoformans* in 1970 [1]. Initially recognized as a variety of *Cryptococcus*, the *Cryptococcus neoformans* var. *gattii* lineage was subsequently elevated to species status as *C*. *gattii* [2]. Further genetic, biochemical, morphological, ecological, and serological characterization of *C*. *gattii* environmental and clinical isolates provided more evidence for the classification of *C*. *gattii* as a unique cryptococcal species [2–10].

The *C*. *gattii* divergence from *C*. *neoformans* is estimated to have occurred 37 to 49 million years ago [11,12]. Since then, *C*. *gattii* has maintained diversity by continuous recombination and evolution into novel lineages with significant genetic diversity that warranted their classification into monophyletic genotypes [12–14]. Recently, the five recognized *C*. *gattii* genotypes, VGI, VGII, VGIII, VGIV, and VGVI were elevated to five individual species: *C*. *gattii*, *C*. *deuterogattii*, *C*. *bacillisporus*, *C*. *tetragattii*, and *C*. *decagattii*, respectively, while the two main lineages of *C*. *neoformans* were raised to species level, becoming *C*. *neoformans* and *C*. *deneoformans* [15]. Different phylogenetic analysis based on concatenated genetic loci unanimously identified *C*. *deuterogattii/*VGII to be the basal lineage of the *C*. *gattii* species complex [11,12,14]. VGI, VGIV, and VGIII diverged from VGII approximately 12.4 million years ago [12,16]. Thereafter, *C*. *tetragattii/*VGIV diverged from *C*. *bacillisporus/*VGIII and *C*. *gattii*/VGI sister clades 11.7 million years ago, finally followed by the split between *C*. *bacillisporus*/VGIII and *C*. *gattii*/VGI 8.5 million years ago.

All seven species of *Cryptococcus* are capable of causing the life-threatening disease cryptococcosis in humans [17]. *C*. *neoformans* (*sensu stricto*), which accounts for 99% of cases worldwide [18–20], typically presents as fungal meningitis in immunocompromised patients. In contrast, infections by the *C*. *gattii* species complex occurs more commonly in otherwise healthy individuals and can often present as fungal pneumonia. During the 20th century, most research considered *C*. *gattii* and *C*. *neoformans* to be interchangeable in their biology. However, the emergence of *C*. *deuterogattii* as the cause of the most devastating and unprecedented fungal outbreak in a healthy human population [21,22] refocused attention on this species, and, as a result, recent research has highlighted key differences between the *C*. *gattii* species complex (CGSC) and *C*. *neoformans* species complex (CNSC). In particular, the apparently low propensity for CGSC species to disseminate from the lung to the central nervous system, and their ability to act as primary pathogens in healthy individuals, remain key unanswered questions [5,23,24].

## Phylogeny and speciation

To date, the majority of *C*. *gattii* studies have focused on *C*. *deuterogattii/*VGII, because of its exceedingly high pathogenicity [21,25] and its role as the predominant etiological agent of the devastating Pacific Northwest Outbreak (PNW) [21,22,26,27]. VGII is not only the cause of the outbreak [27,28] but also possesses a high recombination frequency (via sexual macroevolution and asexual microevolution) producing the highly clonal lineages VGIIa, VGIIb, VGIIc, and VGIIx, which were responsible for the dissemination of the outbreak [21,29–31]. The high clonality of the VGII subtypes has been found to emanate from VGII exhibition of nonclassical same-sex mating, in which sexual reproduction occurs between two alpha mating-type (MATα-MATα) parents [28], which has only been previously described in *C*. *neoformans* [32]. Hence, the PNW outbreak owes its origin and dissemination to this VGII-specific reproductive phenotype.

## Morphological differences between *C*. *gattii* and *C*. *neoformans* species complexes

Most of the virulence-related phenotypic differences between *C*. *gattii* and *C*. *neoformans* are morphological. Within the *Cryptococcus* species complex as a whole, variation in morphological traits such as cell body/capsule size, shape, budding, surface morphology, and cell wall structure and composition are key factors employed not only for the identification of the different cryptococcal lineages [2,33–36], but also to support their elevation to distinct species [15]. In an *in vitro* study of 70 cryptococcal clinical isolates (53 *C*. *neoformans* and 17 *C*. *gattii*), Fernandes and colleagues documented that cellular and capsular enlargement in response to a host-relevant environment is more common in *C*. *gattii* while capsule shedding and production of micro cells were primarily *C*. *neoformans* traits (Table 1) [37]. In the same study, “giant cells” (also known as titan cells; [38]) measuring >15 μm were predominantly found in CGSC rather than in CNSC (50.0% versus 10.75%, respectively), an observation that was recapitulated in a *Drosophila* model of infection [39].

**Table 1:** Comparison of morphological attributes between 70 clinical isolates of cryptococci from HIV/AIDS patients in Botswana-Africa (adapted from [37] and summarized).

Species/genotype (No. of isolates)	Mean Cell diameter (μm)	Mean Capsule thickness (μm)	Giant cells (%)	Micro cells (%)	Shed capsule (%)
*C*. *neoformans/* VNI (17)	7.0	5.5	12	82	94
/ VNII (2)	6.9	3.9	0	0	50
/VNBI (25)	8.3	7.3	20	52	80
/VNBII (9)	7.1	5.3	11	44	67
**All *C***. ***neoformans* (53)**	**7.32**	**5.5**	**10.75**	**44.5**	**72.75**
*C*. *gattii* /VGI (1)	10.2	15.7	50		0
*C*. *tetragattii* /VGIV (16)	9.9	9.3	50	0	0
**All *C***. ***gattii* (17)**	**10.05**	**12.0**	**50**	**0**	**0**

**Fig 1:**
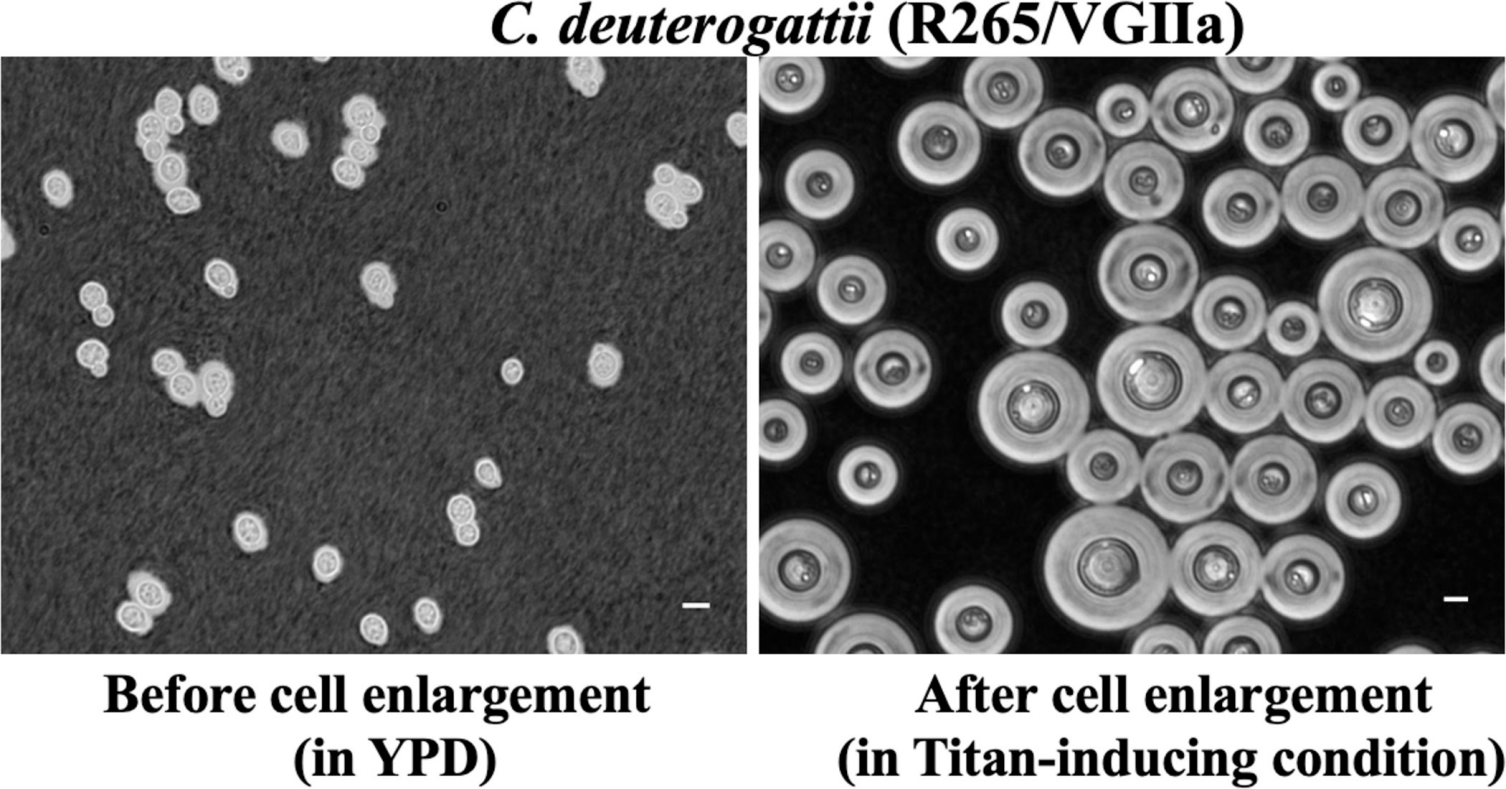
Micrograph showing the budding nature of *C*. *deuterogattii* yeast (left panel) vs. titan (right panel) cells [44]. Scale bar = 5 μm.

Using an *in vitro* titan induction system, we recently showed that the capacity to produce titan cells, an atypical morphotype that is formed when cryptococcal yeast cells transform into extremely enlarged polyploid cells, is more abundant in CGSC than in CNSC [40]. Interestingly, this correlates with a “staggered” cell cycle in *C*. *deuterogattii*, in which cell size increase precedes DNA replication—something that is not seen in *C*. *neoformans* [40]. Whereas *C*. *neoformans* titan cells undergo cell division to produce daughter cells [40–43], CGSC titan cells exhibit a growth arrest to form large unbudded cells [40] (Fig 1). It is possible that this difference may partially explain *C*. *deuterogattii*’s lower ability to disseminate outside the lungs, since *C*. *neoformans* titan cells likely rely on their small-sized daughter cells for dissemination [41,42].

These morphological differences also lead to differences in the host response. For example, the presence of enlarged CGSC cells was associated with high CD4^+^ T cell count, while the formation of small phenotype “micro” cells by CNSC correlates with meningeal irritation and an aggressive inflammatory response [37].

Compositional differences in capsule are also likely to play a major role in varying virulence profiles. While exploring the interaction of *C*. *gattii* with the phagocytic amoebae *Acanthamoeba castellanii*, Malliaris and colleagues [45] discovered a significantly lower phagocytosis profile and reduced virulence of an acapsular cryptococcal mutant strain, *cap67*, when the mutant strain was coated with capsular extract from *C*. *gattii* (serotype B), versus extract from *C*. *neoformans* (serotype A). Although the underlying mechanism is not known, the result suggests the presence of structural difference(s) in *C*. *gattii* capsular polysaccharide, which have a direct impact on virulence. Chemical and biophysical differences in the structure of the major capsular polysaccharides in CGSC versus CNSC have been well documented [46]; consequently, it will be interesting in the future to assess how these biochemical differences relate to the differing host response.

After the polysaccharide capsule, the most important virulence determinant of cryptococci [46,47] is cell wall melanisation [33,48–53]. Melanin is a negatively charged hydrophobic pigment formed by the oxidative polymerization of phenolic compounds [51] and its synthesis is catalysed by laccase. The production of melanin is not only essential for maintaining cell wall integrity but also protects the fungi from environmental stressors, such as UV light and high temperature, and the host immune system [54]. Interestingly, its pattern of distribution varies between strains; for instance, being homogenous in the *C*. *deuterogattii/*VGII hypervirulent outbreak strain CDCR265 [33] but heterogeneously distributed in *C*. *neoformans* H99. Interestingly, in a *Galleria mellonella* infection model, melanization profiles (as determined by laccase activity) of the four *C*. *gattii* molecular types have been directly associated with virulence, such that *C*. *gattii* species complex strains with higher melanin production showed higher lethality towards *Galleria* larvae.

## Immunomodulatory attributes of *C*. *gattii* species complex

The morphological and molecular traits of CGSC discussed above influence how the host immune system responds to infection. The *C*. *deuterogattii* outbreak in the Pacific Northwest (outside its regions of endemicity) that started in 1999 [55] had a mortality rate ranging from 8.7% to 50% even when treated with antifungal drugs [56–61], highlighting significant differences in the host response to this infection.

### Innate immune response to *C*. *gattii* species complex

**Fig 2:**
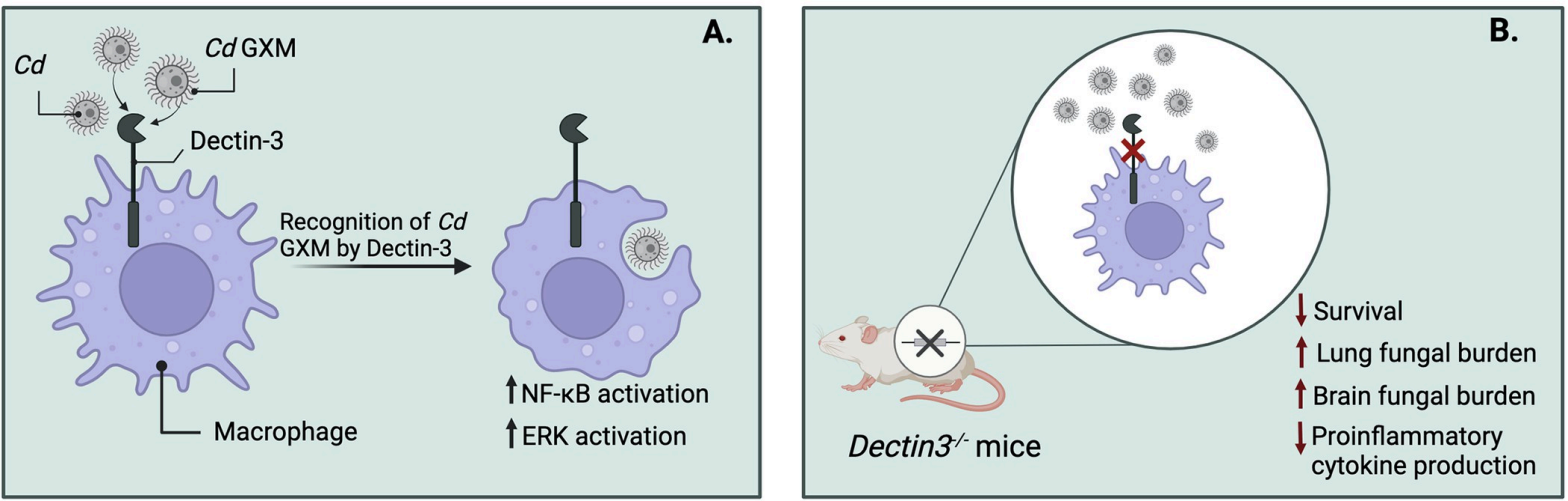
The role of Dectin-3 in host response to *C*. *deuterogattii* infection *in vitro* and *in vivo*. (**A**) The C-type lectin receptor, Dectin-3, recognises *C*. *deuterogattii (Cd)* capsular glucuronoxylomannan (GXM) [64]. The recognition of GXM leads to the activation of NF-κB and ERK signalling pathways to drive proinflammatory cytokine production. (**B**) Dectin-3 deficient mice showed increased susceptibility to *C*. *deuterogattii* infection [64]. Figure created with BioRender.com.

Cryptococcal infection begins with the inhalation of the fungi into the lungs. Lung-resident macrophages are among the first host immune cells that inhaled fungi interact with; however, there are relatively few studies that investigate the precise mechanisms by which phagocytes respond to the presence of CGSC, as opposed to CNSC, in the host. As with *C*. *neoformans*, the capsule of CGSC cells, which is composed of a majority glucuronoxylomannan (GXM) and a minority galactoxylomannan (GalXM) polysaccharide, functions as a fungal virulence factor and has antiphagocytic properties [62]. The phagocytosis of foreign particles is initiated by the recognition of pathogen-associated molecular patterns (PAMPs) by host pattern recognition receptors (PRRs), such as members of the Toll-like receptor (TLR) family and the C-type lectin receptor (CLR) family [63]. GXM from *C*. *deuterogattii* was found to be recognised by Dectin-3, a CLR, ultimately leading to NF-κB and ERK-dependent inflammatory responses (Fig 2A) [64]. In the same study, wild-type and *Dectin3*^*−/−*^ mice were infected with *C*. *deuterogattii* intratracheally, and it was observed that *Dectin3*^*−/−*^ mice had decreased survival, greater lung and brain fungal burden, and decreased TNF-α and IL-6 production (Fig 2B). Thus, engagement of Dectin-3 with *C*. *deuterogattii* GXM may activate a broader anticryptococcal immune response. In another study, HEK293A cells transfected with TLR2/1 and TLR2/6 were able to induce NF-κB activation after stimulation with GXM isolated from five different *Cryptococcus* strains among which were *C*. *gattii* serotype B strain CN23/10.993 (*C*. *deuterogattii/*VGII) and serotype C strain HEC40143 (VGIII/*C*. *bacillisporus*) and *C*. *neoformans* serotype A strains T_1_444 and HEC3393 (VNI) and serotype D strain ATCC 28938 (VNIV) [65,66]. Interestingly, GXM from the *C*. *deuterogattii* strain resulted in the greatest activation of NF-κB, suggesting the existence of structural and immunomodulatory differences between the strains [65] in a way that is reminiscent of the study by Malliaris and colleagues [45] discussed above. The five GXM samples were also used to stimulate nitric oxide (NO) production by RAW264.7 macrophages, and it was found that GXM samples from both CGSC strains were able to induce NO production, while those from the three CNSC strains did not [65].

In a study that sought to define the cytokine profile produced by cryptococcal infection, human peripheral blood mononuclear cells (PBMCs) from healthy individuals were infected with heat-killed CGSC and CNSC clinical isolates. It was found that CGSC isolates induced a greater expression of IL-1β, TNF-α, IL-6, IL-17, and IL-22, compared to CNSC isolates [67]. Meanwhile, there was no difference in IL1Ra levels between the strains. Lastly, it was observed that the modulation of CGSC*-*induced cytokine production required TLR4 and TLR9, but not TLR2.

### Adaptive immune response to *C*. *gattii* species complex

The uptake of fungi into phagosomes and subsequent phagosome maturation results in the degradation of the fungus and the presentation of fungal peptides on major histocompatibility complex (MHC) molecules [68]. These peptides are then recognised by CD4^+^ T cells, thereby activating the adaptive immune response [69]. Additionally, the cytokine profile produced by the activation of PRRs results in the differentiation of naïve T cells into Th1, Th2, or Th17 cells [70]. The Th1 and Th17 responses are known to be protective against *C*. *neoformans*, *Candida albicans*, and *Aspergillus fumigatus* [71–73]. Meanwhile, the Th2 response is anti-inflammatory and promotes fungal survival and dissemination in *C*. *neoformans* [23,68]. It has been shown that mice infected with *C*. *deuterogattii and C*. *gattii* had reduced Th1 and Th17 cells in their lungs compared to those infected with *C*. *neoformans* H99 [24]. This suggests that CGSC is able to successfully infect immunocompetent hosts by dampening the activation of the protective Th1/Th17 response and enhancing the nonprotective Th2 response [24]. The diminished Th1/Th17 response was likely driven by a decrease in the expression of MHC-II on the surface of dendritic cells from *C*. *deuterogattii*- and *C*. *gattii*-infected mice. Huston and colleagues [74] have also shown that dendritic cells infected with *C*. *deuterogattii* fail to exhibit increased expression of MHC-II molecules, CD86, CD83, CD80, and CCR7, which are needed for T cell activation. Therefore, at least two CGSC strains are able to subvert dendritic cell–mediated activation of the adaptive immune response.

Another mechanism by which CGSC is thought to subvert immune recognition is through its capsule polysaccharide. Urai and colleagues [66] found that mice infected with *C*. *deuterogattii/*VGII strain JP02 showed poorer survival and decreased inflammatory cell infiltration when compared to mice infected with *C*. *neoformans* H99. This difference in virulence was attributed to the *C*. *deuterogattii* capsule structure because exposing dendritic cells to purified GXM from JP02 did not induce IL6, IL12p40, and TNF-α production but stimulation with H99 GXM did. This finding is reinforced by a 2021 study by Ueno and colleagues [75] that found that an acapsular *cap60*Δ *C*. *gattii* (VGI) mutant was easily phagocytosed and killed by dendritic cells; however, when capsular polysaccharide from *C*. *gattii* was deposited onto the acapsular mutant, phagocytosis was hindered and IL6 and IL23 proinflammatory cytokine expression was dampened [75]. Therefore, capsular polysaccharide aids immune evasion by shielding *C*. *gattii sensu stricto* from recognition by dendritic cells, thereby preventing the expression of proinflammatory cytokines and hindering clearance of the fungi [75]. This ability to evade immune detection also provides an explanation for why strains within the *C*. *gattii* species complex are able to infect both immunocompetent and immunocompromised individuals while *C*. *neoformans* strains predominantly infect immunocompromised people.

It is important to mention that the result by Urai and colleagues [66] and Ueno and colleagues [75] is in contrast with earlier findings by Fonseca and colleagues [65], which showed that GXM from *C*. *deuterogattii* were the most potent in inducing TLR-mediated NF-κB expression and NO production in macrophages. It also contrasts with results from Schoffelen and colleagues [67], which found that PBMC exposed to CGSC strains produced more proinflammatory cytokines than those exposed to CNSC strains. This may be explained as differences in macrophage versus dendritic cell response. Alternatively, it may represent variation within the CGSC, highlighting the need to be precise about the “*C*. *gattii*” species used. Aside from the within CGSC variation, all these studies point to the existence of significant variation in immune response to CGSC and CNSC infection. As more is understood about host interaction with cryptococcal species, novel therapeutic agents can be developed to decrease the case–fatality rate of infection with cryptococci.

### What are the drivers of the *C*. *deuterogattii* outbreak?

In 1999, an outbreak of *C*. *gattii* (now recognised as *C*. *deuterogattii*) was identified in British Columbia, which went on to become the largest life-threatening primary fungal outbreak in a healthy population. This unprecedented outbreak has driven an intense research effort to discover the underlying determining factors [25,26,76,77]. Over the last decade, studies focused on outbreak strains revealed a hypervirulent *C*. *gattii* molecular type *C*. *deuterogattii/*VGII, to be the primary agent driving the pathogenesis [21,78,79] and spread [28,80] of the outbreak.

*C*. *deuterogattii/*VGII outbreak isolates harbour several unique cellular and genetic attributes that contribute to their hypervirulence and pathogenicity. Compared to other CGSC lineages, VGII outbreak strains display higher resistance to host immune defence mechanisms [78,79] and an overall increased survival profile within the host [79,81]. Upon phagocytosis by macrophages, *C*. *deuterogattii*/VGII responds to the host oxidative burst by triggering rapid intracellular proliferation [78]. A follow-up study revealed that there is in fact a “division of labour” mechanism where a subpopulation of fungal cells undergo growth arrest, thereby facilitating the rapid proliferation of neighbouring fungal cells [79], in a process that is mediated through the exchange of extracellular vehicles (EVs) [81]. Interestingly, the pattern of titan cell formation also differs in this outbreak lineage (discussed above), and it will be interesting to explore and potentially establish the correlation between this unique VGII-titan feature, division of labour responses, and their combined influence on virulence.

In addition to cellular phenotypes, novel genomic traits found in PNW outbreak isolates are thought to contribute to their hypervirulence [16,31]. Genomic analysis among the four VG lineages reveals that, unlike other VG lineages, VGII outbreak isolates have acquired genes encoding proteins involved with membrane trafficking such as Friend of Prmt1 (Fop), and genes involved in heat tolerance such as heat shock protein 70 (HSP70), genes that are known to be required for virulence in *C*. *neoformans* [82]. Perhaps the most striking genomic trait of VGII is the lack of RNA interference (RNAi) machinery due to the absence of genes encoding the Argonaute proteins Ago1 and Ago2 [16], PAZ, Piwi, and DUF1785 genes, which are key regulator of the RNAi machinery in *C*. *neoformans* [31,83]. In fact, analysis from whole genome studies discovered a total of 146 genes (including the RNAi-associated ones) missing in VGII outbreak isolates, which is three times more than those lost in VGI-III/VGIV combined [31]. Although the true significance of the absence of these genes/pathways as a whole is not yet elucidated, it is likely that they contribute to the unique host–pathogen interaction seen in this lineage [28,84].

## Understudied virulence-associated phenotypes of *C*. *gattii*

Studies elucidating the virulence of CGSC phenotypes and their impact on pathogenesis and disease outcomes are somewhat overshadowed by that of *C*. *neoformans* [76]. Often, the biology, virulence factors, and pathogenicity of *Cryptococcus* are highlighted using *C*. *neoformans* as a model or the primary theme of study [46,52,85,86]. Below are some examples of understudied CGSC virulence phenotypes.

### Capsule

The cryptococcal polysaccharide capsule is essential for both cellular function and pathogenesis of *Cryptococcus*, protecting the fungus against desiccation in the environment and playing the synergistic role of a shield and virulence mechanism in animal host [46,47]. Thus, the capsule is considered the most important virulence determinant of cryptococci. Evidence has shown that capsular size and its impact on host immune response in the *C*. *gattii* lineage differs from that of *C*. *neoformans* [37,84]. Despite these differences in capsule properties, the biosynthesis properties [18], biophysical properties [46,47,87,88], and immunogenic properties [89] of cryptococcal capsule have primarily been characterized in *C*. *neoformans*. Although the physical impact of capsule on fungal biology is likely to be similar for both species, the impact of varying capsule composition on CGSC virulence remains less well understood [90].

### Morphogenesis

The most dramatic host adaption phenotype exhibited by *Cryptococcus* is the formation of titan cells [43,91,92], a phenomenon that has thus far been largely studied in *C*. *neoformans* [92–96]. We and others have recently investigated titan cell formation across the broader *Cryptococcus* species complex, which has revealed some subtle differences between lineages [44]. The impact of these differences on the CGSC–host interaction remains largely unknown [97–99]. Similarly, while several signalling pathways, receptors, and genes have been identified as driving titan cell formation in *C*. *neoformans* (including the cyclic AMP (cAMP)/protein kinase A (PKA) pathway (putatively associated with *C*. *neoformans* pathogenesis), G protein-coupled receptors (Gpr4 and Gpr5), St3a [38], and the CLN1 genes [93]), such investigations are yet to be performed with *C*. *gattii* and its sister species.

### Extracellular vesicles

Extracellular vesicles (EVs) are rounded bilayered particles produced by prokaryotic and eukaryotic cells to mediate intercellular communication by transferring information between cells [100]. EVs have significant roles in the cellular and pathogenic lifestyle of cells including stress response, intercellular competition, lateral gene transfer (via RNA or DNA), pathogenicity, and detoxification [101]. The first fungal EV was described in *C*. *neoformans*, and, therefore, the biology of EVs and its role in *C*. *neoformans* virulence is well studied and documented [102–105]. Cryptococcal EVs are carriers of several virulence molecules such as capsular GXM, laccase, urease, and a repertoire of immunogenic proteins and thus have been termed “virulence bags” [104,105].

The biological and functional features of EVs including biosynthesis, secretory pathways, composition, structure, virulence properties and mechanism, influence in pathogen–host interaction, and immunogenic-related attributes have been well characterized in *C*. *neoformans* [102,104–107]. However, our knowledge and concepts of *C*. *neoformans-*derived EVs cannot be directly applied to CGSC strains, since evidence has shown distinctive features of EVs in the two species complexes [102]. For instance, the size of *C*. *deuterogattii* EVs is significantly smaller than *C*. *neoformans* and *C*. *deneoformans* [102]. Although homologous EV-protein families, such as the putative glyoxal oxidase (Gox proteins) and Ricin-type beta-trefoil lectin domain-containing protein (Ril), were characterized in all three species, the Sur7/Pal1 family of tetraspanin membrane proteins was exclusive to *C*. *deuterogattii* [102]. Moreover, two of the ferroxidase Cfo proteins investigated were found to be expressed only by *C*. *deuterogattii* EVs but not in the two other species [108].

In terms of function, the novel discovery of *C*. *deuterogattii* EV-based long-distance pathogen-to-pathogen communication (which has not been reported for non-*gattii* strains) [81], its role in exploiting host immune cells, and ultimate impact on virulence [79,81] provide strong evidence that the functional mechanism of EVs differs in the two lineages. This discovery and other findings on CGSC-specific EV traits highlight the need for probing the already-studied concepts of *C*. *neoformans-*derived EV and novel EV-associated phenotypes in these other species. Research focused on CGSC EVs will not only diversify our understanding of cryptococcal EVs but also holds potential for revealing new paradigms around the biological and pathogenic functions of fungal EVs more broadly.

We conclude this section with a list of *C*. *neoformans* phenotypic virulence traits that are somewhat neglected and could potentially be studied in *C*. *gattii* (Table 2).

**Table 2:** List of virulence-related phenotypic traits whose underlying molecular, genetic, and metabolic mechanism has been studied in *C*. *neoformans* but not *C*. *gattii*.

Virulence factor	Virulence factor regulatory genes/pathways	Mode of action/functional mechanism	References
Capsule	*Cap59* gene	Encodes transmembrane proteins and is involved in GXM synthesis	[109], [110,111]
	*Cap64* gene	Complements an acapsular strain and is required for capsule synthesis	[112]
	*Cap60 gene*	Encodes proteins localized to the nuclear membrane and cytoplasm; required for both capsule formation and virulence	[113]
	*Uge1p* gene; putative UPD-galactose epimerase	Required for GalXM synthesis and consequent crossing of the blood–brain barrier	[114]
	Putative G1/S cyclin (Cln1)	Regulates the cell cycle during capsule formation; required for virulence at 37°C	[115]
	Rim101 transcription factor via cAMP/PKA pathways	Required for polysaccharide attachment to the cell wall surface	[116]
	CLN1 gene	Encodes *C*. *neoformans* cyclin Cln1; critical for balancing DNA replication and cell division during titan cell formation; negatively regulates *in vivo* titan cell formation	[93]
Titan cell formation	Rim101 transcription factor via cAMP/PKA pathways	Required for the generation of titan cells	[117]
	Usv101 transcription factor, GPA1, CAC1, Ric8, and PKA1 genes associated with cAMP/PKA pathway	Negatively regulates titan cell formation *in vivo* and *in vitro*	[118] [41] [117]
	G1/S cyclin (Cln1)	Required for cell wall stability and production of melanin; protective against oxidative damage; positively regulates the production of laccase	[119]
Cell wall	Proteinase	Proteolytic activity against host proteins including collagen, elastin, fibrinogen, immunoglobulins, and complement factors	[120]
Degradative enzymes (proteinase, phospholipases, urease)	Phospholipase enzyme (PLB1 gene)	Regulates phospholipase B (PLB), lysophospholipase hydrolase, and lysophospholipase transacylase activities; positively regulates virulence *in vivo* and is required for intracellular growth and vomocytosis	[52] [121]
	Urease (Ure1 gene)	Required for virulence in mice, CNS invasion; and vomocytosis	[122] [121]
	CNA1 gene	Encodes *C*. *neoformans* calcineurin A; required for growth at mammalian physiological temperature; required for virulence in immunocompromised animal model	[40]

## Concluding remarks

**Fig 3:**
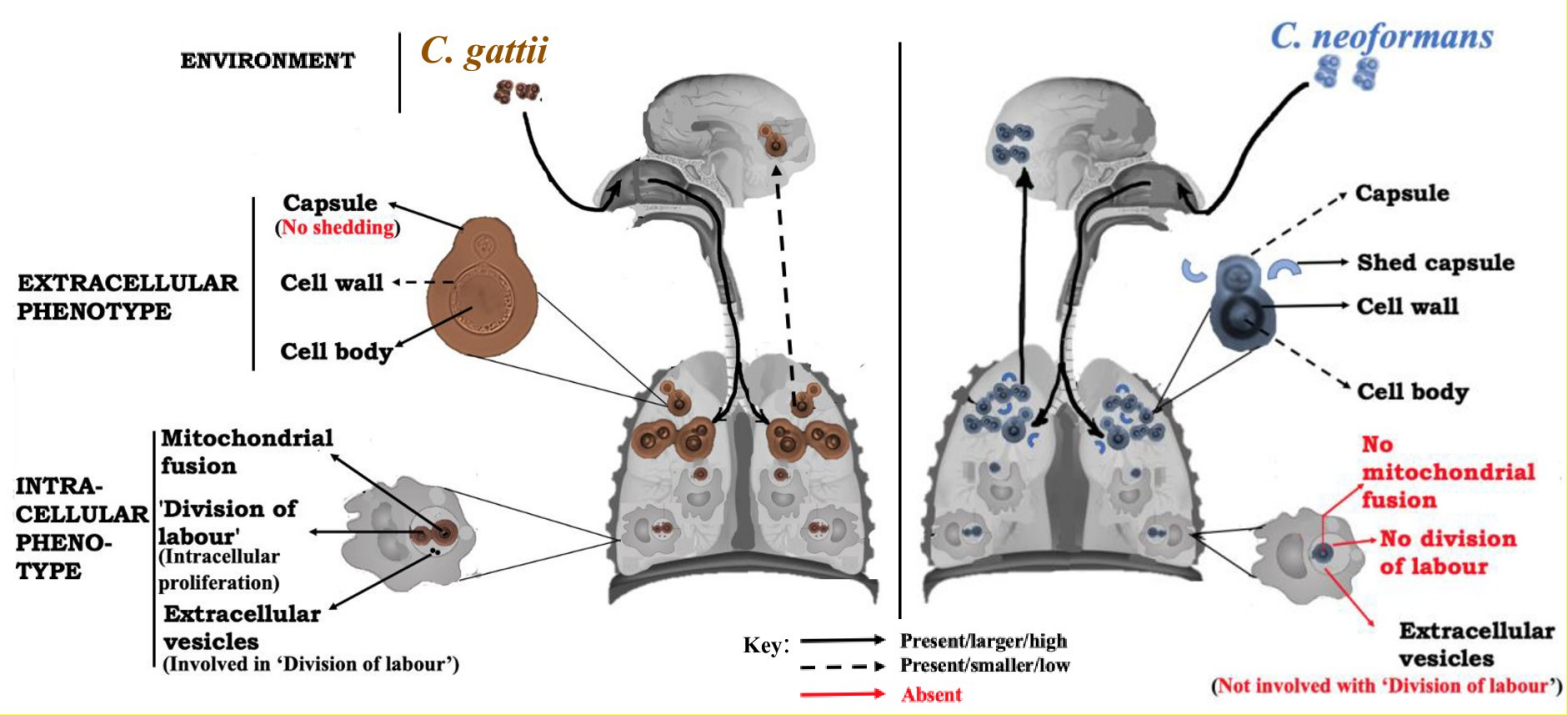
Schematic diagram illustrating CGSC distinct phenotypic virulence traits as compared to *C*. *neoformans*. Upon inhalation from the environment, CGSC yeast cells/spores responds to the lung extracellular niche by exhibiting phenotypic traits including larger capsule (with less immunogenic properties), larger cell body (with higher degree of homogeneity), and thinner but more compacted cell wall with higher chitosan content than *C*. *neoformans*. The manner in which CGSC strains exhibit these host-adaptive traits is perhaps responsible for its low affinity to dissemination from the lungs to the brain. Within the host macrophage, the intracellular phenotypes (mitochondrial fusion and “division of labour” proliferation mechanism mediated by extracellular vesicles) exhibited by *C*. *deuterogattii* (which drives the fatal Pacific Northwest outbreak) are absent in *C*. *neoformans*.

Despite their similar appearance, it is now clear that many features of the CGSC are remarkably different from those in the CNSC (Fig 3). However, studies elucidating the virulence of *C*. *gattii* phenotypes and their impact on pathogenesis and disease outcomes have been overshadowed by those focusing on CNSC. While the prevalence of cryptococcosis due to CNSC makes this a logical choice, several unique features of *C*. *gattii* and its sister species warrant further investigation, including their ability to infect otherwise healthy individuals, their role in an unprecedented fungal outbreak and several distinct phenotypic traits that differ from those in *C*. *neoformans*. Such features are likely to underlie important differences in clinical presentation between the two pathogens, most notably in their varying patterns of dissemination from the lungs to the brain. In the future, it will be important to revisit many paradigms of cryptococcal pathogenesis in CGSC strains and, indeed, other *Cryptococcus* species and, in doing so, reveal the full range of diversity within this genus.

**Key Learning Points**

- Although morphologically similar, members of the *Cryptococcus gattii* species complex and *Cryptococcus neoformans* species complex exhibit important differences in biology and pathogenesis.
- Their interaction with the human immune system is one such key difference, with evidence for more profound immune-dampening mechanisms within the *C*. *gattii* species complex, contributing towards its enhanced ability to infect healthy hosts.
- Both species complexes form titan cells, but the triggers and mechanisms by which they do so are subtly different.
- Extracellular vesicle release occurs in both species, but only *C*. *deuterogattii* has thus far been shown to use these vesicles to coordinate a virulence strategy.
- The recent recognition that lineages within both species are sufficiently different to warrant elevation to species level should serve as a prompt for a more detailed examination and appreciation of the varying biology within this genus.

**Top Five Papers**

1. Byrnes EJ, 3rd, Li W, Lewit Y, Ma H, Voelz K, Ren P, et al. Emergence and pathogenicity of highly virulent Cryptococcus gattii genotypes in the northwest United States. PLoS Pathog. 2010;6(4):e1000850. Epub 2010/04/28. doi: 10.1371/journal.ppat.1000850. PubMed PMID: 20421942; PubMed Central PMCID: PMC2858702.
2. Hagen F, Khayhan K, Theelen B, Kolecka A, Polacheck I, Sionov E, et al. Recognition of seven species in the Cryptococcus gattii/Cryptococcus neoformans species complex. Fungal Genet Biol. 2015;78:16–48. Epub 2015/02/28. doi: 10.1016/j.fgb.2015.02.009. PubMed PMID: 25721988.
3. Kidd SE, Hagen F, Tscharke R, Huynh M, Bartlett KH, Fyfe M, et al. A rare genotype of Cryptococcus gattii caused the cryptococcosis outbreak on Vancouver Island (British Columbia, Canada). Proceedings of the national academy of sciences. 2004;101(49):17258–63.
4. Voelz K, Johnston SA, Smith LM, Hall RA, Idnurm A, May RC. ‘Division of labour’in response to host oxidative burst drives a fatal Cryptococcus gattii outbreak. Nat Commun. 2014;5(1):1–12.
5. Cheng PY, Sham A, Kronstad JW. Cryptococcus gattii isolates from the British Columbia cryptococcosis outbreak induce less protective inflammation in a murine model of infection than Cryptococcus neoformans. Infect Immun. 2009;77(10):4284–94. Epub 2009/07/29. doi: 10.1128/iai.00628-09. PubMed PMID: 19635827; PubMed Central PMCID: PMC2747943.
